# Supplementary material for: An ancient metabolite damage-repair system sustains photosynthesis in plants
Source: Nat Commun. 2023 May 25;14:3023. doi: 10.1038/s41467-023-38804-y (PMC10212915; doi:10.1038/s41467-023-38804-y)
Supplement: Supplementary file 1 — Supplementary Information [file 41467_2023_38804_MOESM1_ESM.pdf]

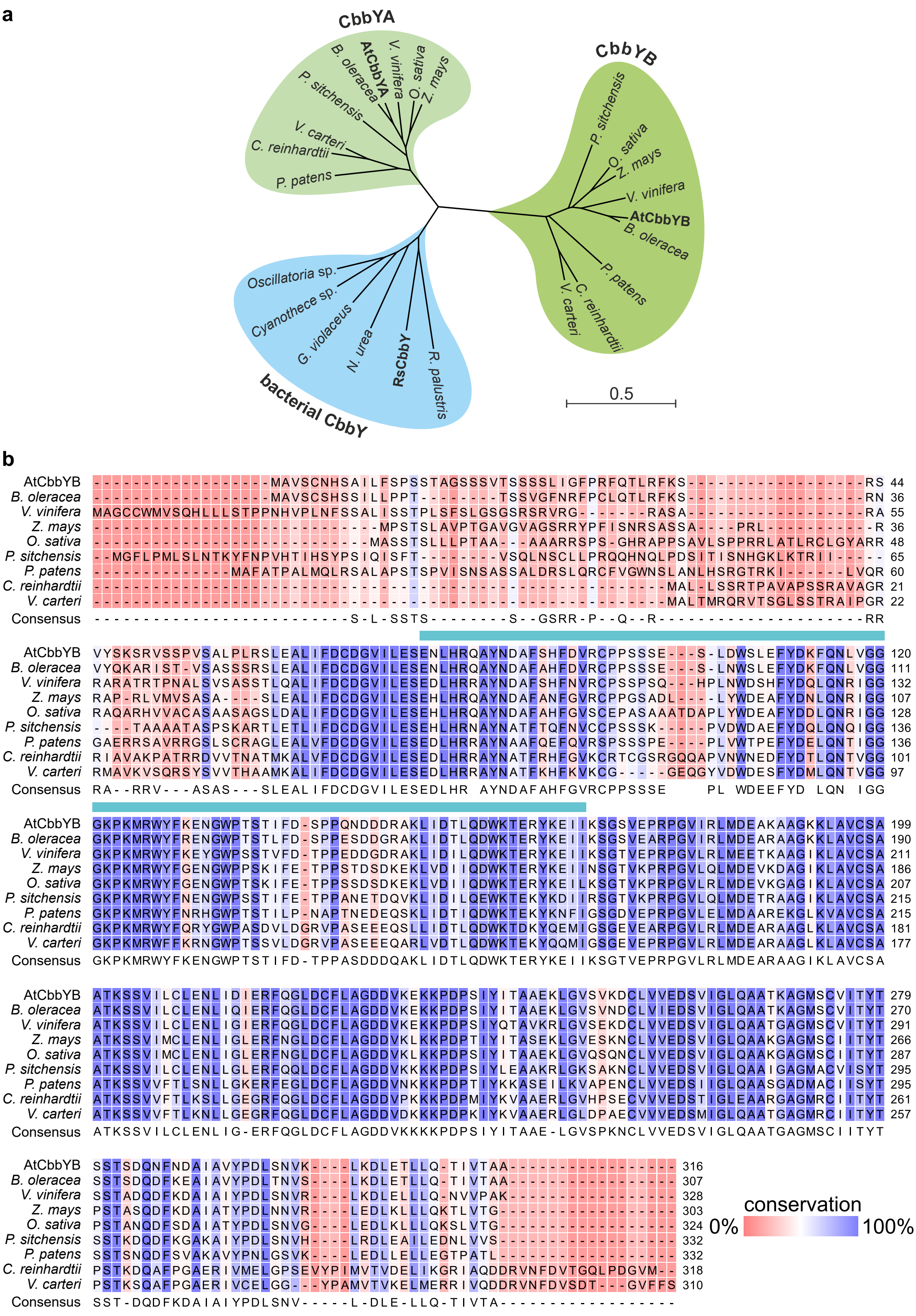

Supplementary Figure 1 Molecular phylogenetic analysis of CbbY proteins.
a The evolutionary history of CbbY proteins was inferred by using the Maximum Likelihood method. The tree is drawn to scale, with branch lengths measured in the number of substitutions per site according to the Whelan and Goldman method. The analysis involved 24 amino-acid sequences (see Supplementary Table 1 for identifiers). A bootstrap analysis was performed with 1000 replicates and bold lines indicate bootstrap values >50%. **b** Multiple sequence alignment of CbbYB proteins. Conservation is depicted on a color scale from red (0%) to blue (100%). The cap domain is labeled by a turquoise bar above the sequences. Alignment and evolutionary analyses were carried out with the CLC Workbench Software (version 20). RsCbbY, CbbY of *Rhodobacter sphaeroides*; AtCbbYA, CbbYA of Arabidopsis; AtCbbYB, CbbYB of Arabidopsis.

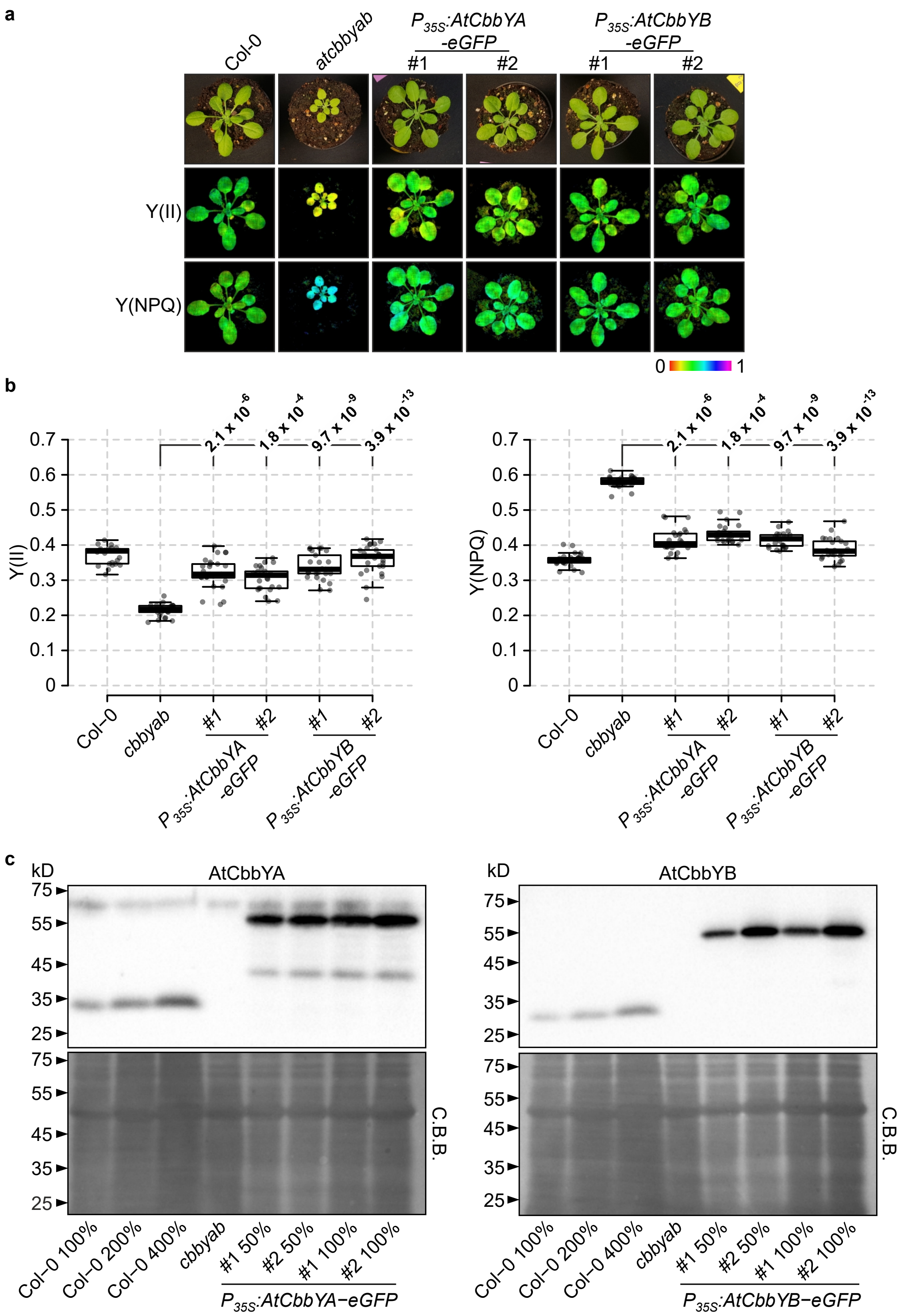

Supplementary Figure 2 Complementation analyses.
a Phenotype restoration was examined using two independent lines, each transformed with either the *P_{35S}:AtCbbYA-eGFP* or *P_{35S}:AtCbbYB-eGFP* construct. The effective quantum yield of photosystem II [Y(II)] and the yield of non-photochemical quenching [Y(NPQ)] were determined in a light induction experiment (600 s after exposure to 185 µmol photons m^{-2} s^{-1}) by Imaging-PAM. Parameters are visualized on a false-color scale from zero to one, which is shown at the bottom of the panel. **b** Quantification of Y(II) and Y(NPQ) parameters. Four leaves from each of five plants per genotype were examined (n=20). Center lines show the medians, and boxes indicate the 25th and 75th percentiles. Whiskers denote 1.5x the interquartile range. Data points are plotted as grey circles and outliers are represented by dots. For statistical analyses, the non-parametric Kruskal-Wallis test was performed, followed by pairwise Dunn's tests. The *p*-values were adjusted on an experiment level using the Benjamini-Hochberg method. *P*-values for comparison of complemented lines with *atcbbyab* are indicated and $p \leq 0.05$ are marked in bold. **c** Amounts of AtCbbYA-eGFP and AtCbbYB-eGFP were analyzed by immunodetection. Leaf protein extracts of Col-0 and *atcbbyab* were loaded as references in different dilutions as indicated (100% corresponded to protein extract isolated from 2 mg leaf fresh weight). As a loading control, PVDF membranes were stained with Coomassie Brilliant Blue 250G (C.B.B.). Immunodetections were carried out two times with similar results.

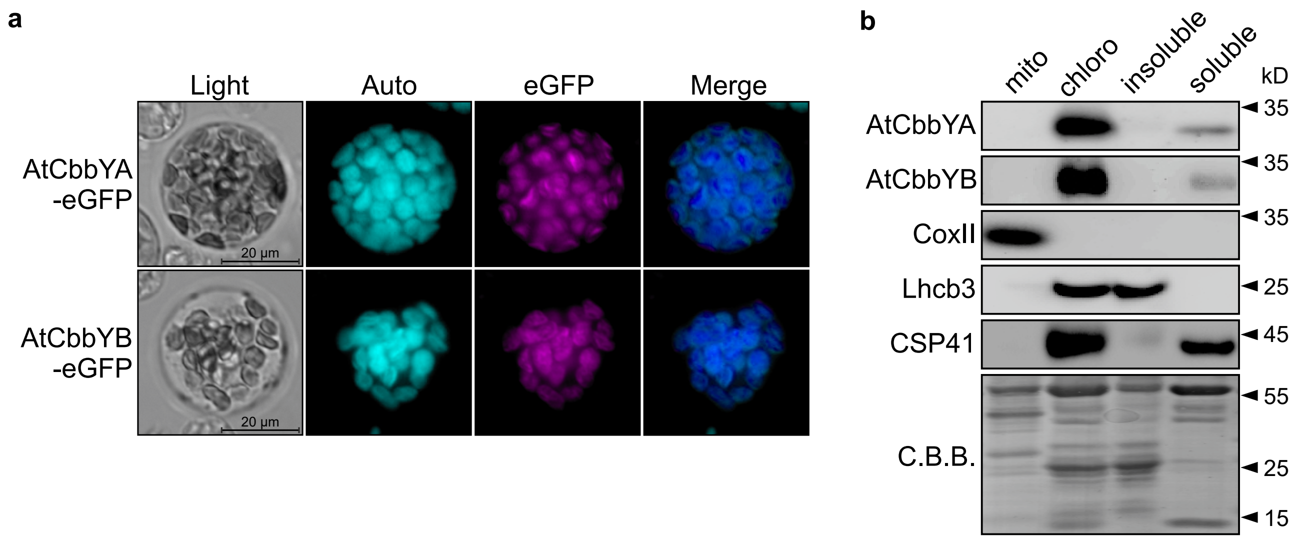

Supplementary Figure 3 Chloroplast localization of AtCbbYA and AtCbbYB.
a Subcellular localization of AtCbbYA-eGFP and AtCbbYB-eGFP was examined in protoplasts isolated from stably transformed plants (P_{35S}:*AtCbbYA-eGFP* and P_{35S}:*AtCbbYB-eGFP*) by fluorescence microscopy. Chlorophyll autofluorescence (Auto) and fluorescence emitted by the enhanced green fluorescence protein (eGFP) were merged (Merge) in one picture. Protoplast integrity was verified by bright-field microscopy (Light). **b** Fractionation and subsequent immunodetection assays of AtCbbYA and AtCbbYB. Mitochondria (mito) and chloroplasts (chloro) were isolated from wild-type plants (Col-0), and chloroplasts were further separated into an insoluble and a soluble fraction. The purity of the fractions was assessed by immunodetection of the indicated marker proteins. CoxII served as a mitochondrial marker, Lhcb3 as a marker for thylakoid localization and CSP41 as a stromal marker protein. As a loading control, PVDF membranes were stained with Coomassie Brilliant Blue G-250 (C.B.B.). Fractionation and immunodetections were performed once.

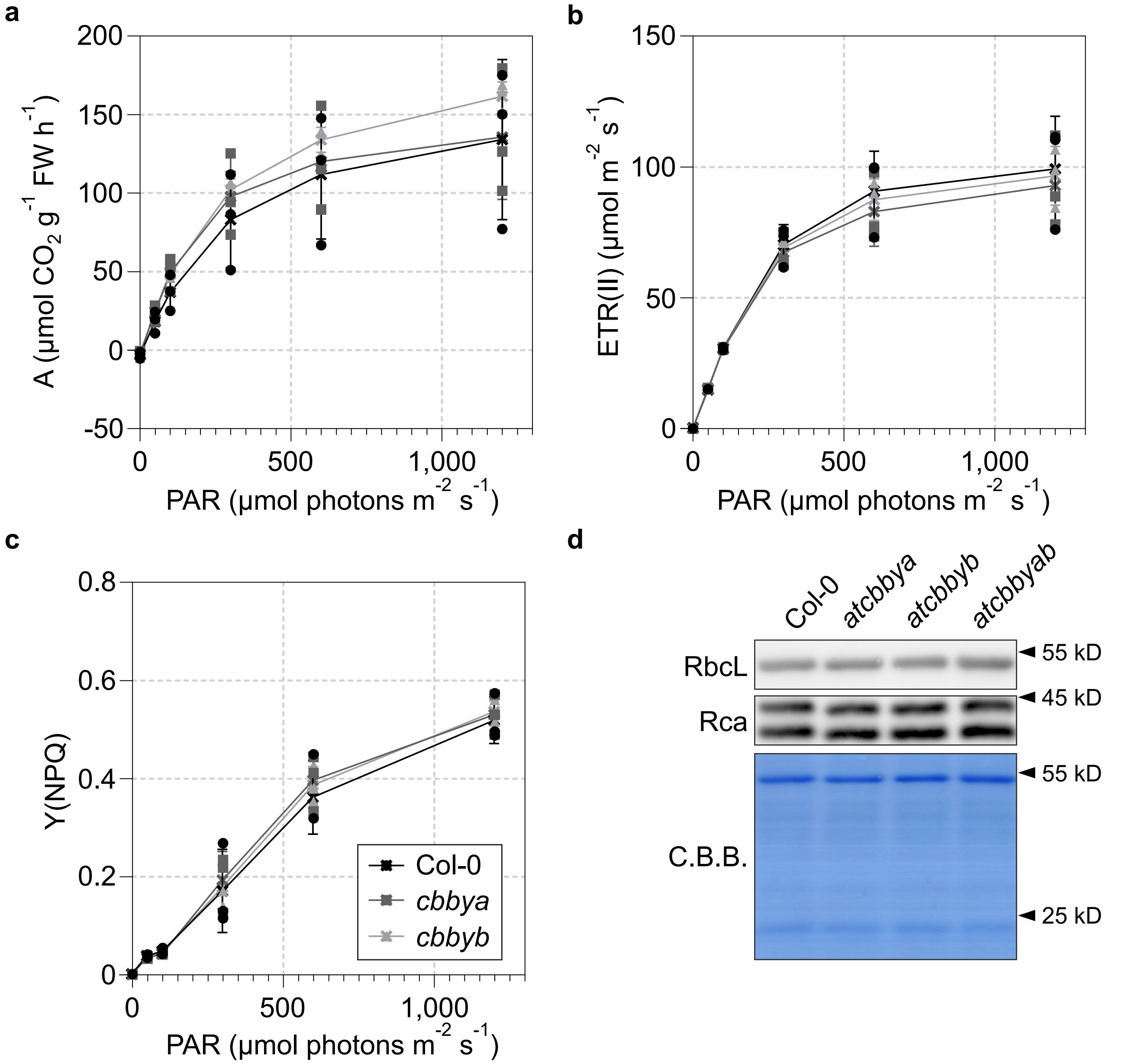

Supplementary Figure 4 CO_2 assimilation and photosynthesis in the single mutants *atcbbya* and *atcbbyb*.

CO_2 assimilation (A) and chlorophyll *a* fluorescence of the single mutants *atcbbya* and *atcbbyb* were quantified with a gas-exchange and fluorescence system GFS-3000 (Walz®, Effeltrich, Germany). **a** CO_2 assimilation rates were analyzed at various light intensities (0, 50, 100, 300, 600 and 1200 µmol photons m^{-2} s^{-1}) in the presence of an ambient CO_2 concentration of 46 Pa in the measuring chamber. **b** Electron transport rates through PSII [ETR(II)]. **c** Non-photochemical quenching [NPQ] parameters. Means (crosses) and standard deviations are provided, which were calculated based on the analysis of three individual plants per genotype (*a-c*). **d** Immunodetection of the large subunit of Rubisco RbcL and Rubisco activase (Rca). Proteins isolated from rosette leaves were size-fractionated by SDS-PAGE and blotted onto PVDF membranes. After staining the PVDF membrane with Coomassie Brilliant Blue G-250 (C.B.B.) to control for equal loading, RbcL and Rca were immunodetected with specific antibodies from Agrisera (the Rca antibody AS10 700 and the RbcL antibody AS03 037 were used at a dilution of 1:5000). Immunodetections were repeated twice with similar results. For each experiment proteins were extracted from pooled leaves of three individual plants.

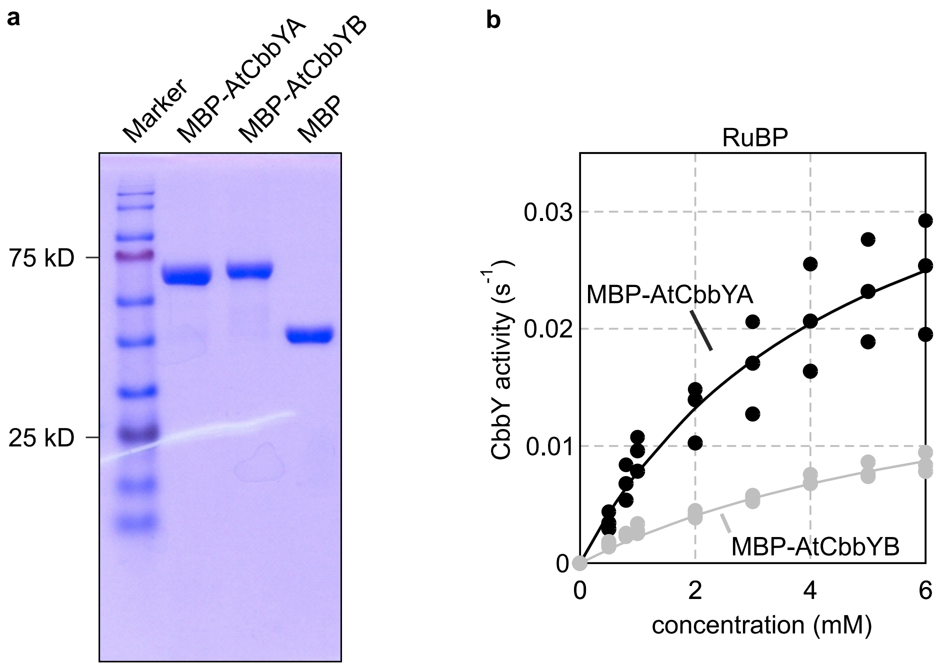

Supplementary Figure 5 Purification of MPB-AtCbbY fusion proteins.

a MBP-AtCbbYA, MBP-AtCbbYB and MBP were purified under native conditions after heterologous expression in BL21 (DE3) *Escherichia coli* cells. A molecular mass standard (Marker) and elution fractions were loaded onto SDS-PAGE, size-fractionated and visualized by Coomassie-staining. **b** Michaelis-Menten plots for MBP-AtCbbYA and MBP-AtCbbYB with RuBP as substrate. Note that the same results are presented in Fig. 5b but with a fitted scaling of the y-axis for comparison to enzyme kinetics assays with XuBP. Three measurements were conducted in each experiment.

Supplementary Table 1 Sequence identifiers of CbbY proteins used in the phylogenetic tree shown in Supplementary Fig. 1.

No	Organism	Protein family	Identifier
1	*Arabidopsis thaliana*	CbbYA	Q94K71
2	*Brassica oleracea*	CbbYA	A0A0D3DRC9
3	*Vitis vinifera*	CbbYA	D7SHD2
4	*Zea mays*	CbbYA	B4FF48
5	*Oryza sativa*	CbbYA	Q10I42
6	*Picea sitchensis*	CbbYA	ABR17576.1
7	*Physcomitrella patens*	CbbYA	Pp3c10_2693V3.1
8	*Chlamydomonas reinhardtii*	CbbYA	A8IX81
9	*Volvox carteri*	CbbYA	XP_002950344
10	*Arabidopsis thaliana*	CbbYB	Q680K2
11	*Brassica oleracea*	CbbYB	A0A0D3BJM8
12	*Vitis vinifera*	CbbYB	F6HJK9
13	*Zea mays*	CbbYB	B4FZG6
14	*Oryza sativa*	CbbYB	Q6ZDS0
15	*Picea sitchensis*	CbbYB	ABR17576.1
16	*Physcomitrella patens*	CbbYB	Pp3c5_20370V3.1
17	*Chlamydomonas reinhardtii*	CbbYB	A8J8H2
18	*Volvox carteri*	CbbYB	XP_002954499
19	*Rhodobacter sphaeroides*	bacterial CbbY	P95649
20	*Rhodopseudomonas palustris*	bacterial CbbY	WP_011473337
21	*Nitrosomonas ureae*	bacterial CbbY	A0A0S3AMM5
22	*Gloeobacter violaceus*	bacterial CbbY	Q7NF42
23	*Cyanothece sp.* PCC 7822	bacterial CbbY	WP_013323490
24	*Oscillatoria sp.* PCC 10802	bacterial CbbY	WP_017721923

Supplementary Table 2 HPLC pigment analysis of Col-0, *atcbbya*, *atcbbyb* and *atcbbyab* plants. Values represent means ± standard deviations calculated from five biological replicates. A biological replicate represents leaves from one individual plant. Pigment amounts were referred to milligram leaf fresh weight (pmol mg^{-1} FW). The pool of violaxanthin, antheraxanthin and zeaxanthin is abbreviated as VAZ, and the ratio of chlorophyll *a* and *b* is expressed as Chl *a/b*. For statistical analyses, the non-parametric Kruskal-Wallis test was performed, followed by pairwise Dunn's tests. The *p*-values were adjusted on an experiment level using the Benjamini-Hochberg method. *P*-values for comparison of mutant lines with Col-0 are indicated.

	Col-0	*atcbbya*	*p*-value	*atcbbyb*	*p*-value	*atcbbyab*	*p*-value
Neoxanthin	44.6 ± 5.5	45.6 ± 4.9	0.807	45.0 ± 4.1	0.708	47.0 ± 7.6	1.0
Violaxanthin	38.6 ± 4.6	43.8 ± 5.5	0.135	42.2 ± 5.9	0.193	51.2 ± 7.5	0.02
Antheraxanthin	1.6 ± 0.5	1.0 ± 0.0	0.146	1.4 ± 0.5	0.333	12.4 ± 1.3	0.021
Lutein	139.6 ± 23.4	155.2 ± 15.9	0.180	163.4 ± 17.8	0.163	182.2 ± 27.1	0.023
Zeaxanthin	0 ± 0	0.0 ± 0.0	0.6	0.0 ± 0.0	0.75	8.4 ± 0.9	0.001
Chlorophyll *a*	1195.6 ± 156.5	1231.8 ± 121.5	1.0	1228.2 ± 115.0	0.782	1197.2 ± 197.9	0.498
Chlorophyll *b*	355.6 ± 48.4	373.8 ± 37.2	1.0	370.8 ± 32.2	0.47	382.0 ± 62.5	0.545
Carotenoids	110.8 ± 16.5	118.6 ± 11.3	0.687	116.6 ± 10.7	0.484	112.6 ± 18.5	0.425
VAZ	40.2 ± 4.6	45.2 ± 6.0	0.232	43.4 ± 6.3	0.302	72.2 ± 9.3	0.004
Chl *a+b*	1551.2 ± 204.6	1605.6 ± 158.4	1.0	1599.0 ± 146.7	0.681	1579.4 ± 260.8	0.502
Chl *a/b*	3.36 ± 0.1	3.3 ± 0.0	0.061	3.31 ± 0.0	0.144	3.13 ± 0.0	0.001

Supplementary Table 3 Primers used in this study.

Primer name	Primer sequence 5' to 3'	Comment
cbbya_RP	AATTTGCAACAATGGTCAAGC	Genotyping, SALK_025204, *AT3G48420* insertion
cbbya_LP	TTCAAATGAGAAGGCGGTATG	Genotyping, SALK_025204, *AT3G48420* insertion
cbbyb_RP	CAGGCAAGTGACCTGAAGAGT	Genotyping, SM_3.15345, *AT4G39970* insertion
cbbyb_LP	ATTCATGGACCACGCCCTTT	Genotyping, SM_3.15345, *AT4G39970* insertion
spm32	TACGAATAAGAGCGTCCATTTTAGA GTGA	John Innes Enhancer trap lines, border primer
LbB1.3	ATTTTGCCGATTTCGGAAC	left border primer SALK lines
At3g48420_Nor_s	AAGGACGGTCACAGGATCTC	Northern probe, *AT3G48420*
At3g48420_Nor_as	GTCTCCTGCGAATATCTTGA	Northern probe, *AT3G48420*
At4g39970_Nor_s	ATGCTTTCTCGCATTTCGATGTTC	Northern probe, *AT4G39970*
At4g39970_Nor_as	CCCTGCAAGGAAGCAATCAA	Northern probe, *AT4G39970*
GW_At3g48420_s	GGGGACAAGTTTGTACAAAAAAGCA GGCTCAATGGCCACTGTGAAAATC	Gateway cloning, *AT3G48420* in pB7FWG2
GW_At3g48420(-Stop)_as	GGGGACCACTTTGTACAAGAAAGCT GGGTTACTAACGAACTGTTTCCG	Gateway cloning, *AT3G48420* in pB7FWG2
GW-At4g39970_s	GGGGACAAGTTTGTACAAAAAAGCA GGCTCAATGGCGGTTTCTTGCAAC	Gateway cloning, *AT4G39970* in pB7FWG2
GW-At4g39970(-Stop)_as	GGGGACCACTTTGTACAAGAAAGCT GGGTTAGCTGCAGTGACTATTGT	Gateway cloning, *AT4G39970* in pB7FWG2
GW-RsCbbY_opt_for	GGGGACAAGTTTGTACAAAAAAGCA GGCTCAATGGCCACTGTGAAAATC	Gateway cloning, *RsCbbY* in pB7FWG2
GW-RsCbbY_opt_rev_EGFP	GGGGACCACTTTGTACAAGAAAGCT GGGTTAGCCACAGGAGCCGTAAG	Gateway cloning, *RsCbbY* in pB7FWG2
RsCbbY_opt_seq_for	ACCACTCCCGATAGCCGACA	Sequencing primer for pB7FWG2-RsCbbY
RsCbbY_opt_seq_rev	CGCCTCAACATTCGGTAAAG	Sequencing primer for pB7FWG2-RsCbbY
At3g48420_pET151_s	CACCTGCTCTGCTTCATCTTCTCT	protein synthesis, 6xHis-tagged, pET151 D-TOPO
At3g48420_pET151_as	TTAACTAACGAACTGTTTCCGGA	protein synthesis, 6xHis-tagged, pET151 D-TOPO
At4g39970_pET101_s	CACCATGTCGAAATCTAGAGTTTCTT CTCCTGTC	protein synthesis, 6xHis-tagged, pET101 D-TOPO
At4g39970_pET101_as	AGCTGCAGTGACTATTGTTTGAAGC	protein synthesis, 6xHis-tagged, pET101 D-TOPO
MBP_CbbYA_rev	GGTCCTGAATTCTTAACTAACGAACT GTTTCCGGAGA	MBP fusion, in pMal-c5x
MBP_CbbYA_for	TGCTCTGCTTCATCTTCTCTGACGA	MBP fusion, in pMal-c5x
MBP_CbbYB_rev	GGTCCTGAATTCTTAAGCTGCAGTG ACTATTGTTTGA	MBP fusion, in pMal-c5x
MBP_CbbYB_for	TCGAAATCTAGAGTTTCTTCTCCTGT C	MBP fusion, in pMal-c5x
