# Supplementary material for: An ancient metabolite damage-repair system sustains photosynthesis in plants
Source: Nat Commun. 2023 May 25;14:3023. doi: 10.1038/s41467-023-38804-y (PMC10212915; doi:10.1038/s41467-023-38804-y)
Supplement: Supplementary file 3 — Description of Additional Supplementary Files [file 41467_2023_38804_MOESM3_ESM.pdf]

Description of Additional Supplementary Files:

Supplementary Data 1. AT3G48420 (AtCBBYA) co-expressed genes. Data were obtained from the ATTED database (https://atted.jp/).

Supplementary Data 2. Functional profiling of AT3G48420 (AtCBBYA) co-expressed genes. The analysis was conducted using g:Profiler (https://biit.cs.ut.ee/gprofiler/gost). The significance threshold was set to 10-5. The method for multiple testing correction followed the Bonferroni correction. Analysis was carried out in ordered query according to the ranking of AT3G48420 (AtCBBYA) co-expressed genes.
