# Supplementary material for: An ancient metabolite damage-repair system sustains photosynthesis in plants
Source: Nat Commun. 2023 May 25;14:3023. doi: 10.1038/s41467-023-38804-y (PMC10212915; doi:10.1038/s41467-023-38804-y)
Supplement: Supplementary file 6 — Reporting Summary [file 41467_2023_38804_MOESM6_ESM.pdf]

nature portfolio

Corresponding author(s): Thilo Ruehle

Last updated by author(s): May 8, 2023

Reporting Summary

Nature Portfolio wishes to improve the reproducibility of the work that we publish. This form provides structure for consistency and transparency in reporting. For further information on Nature Portfolio policies, see our Editorial Policies and the Editorial Policy Checklist.

Statistics

For all statistical analyses, confirm that the following items are present in the figure legend, table legend, main text, or Methods section.

n/a	Confirmed	
☐	☒	The exact sample size (*n*) for each experimental group/condition, given as a discrete number and unit of measurement
☐	☒	A statement on whether measurements were taken from distinct samples or whether the same sample was measured repeatedly
☐	☒	The statistical test(s) used AND whether they are one- or two-sided<br>*Only common tests should be described solely by name; describe more complex techniques in the Methods section.*
☒	☐	A description of all covariates tested
☐	☒	A description of any assumptions or corrections, such as tests of normality and adjustment for multiple comparisons
☐	☒	A full description of the statistical parameters including central tendency (e.g. means) or other basic estimates (e.g. regression coefficient) AND variation (e.g. standard deviation) or associated estimates of uncertainty (e.g. confidence intervals)
☐	☒	For null hypothesis testing, the test statistic (e.g. *F*, *t*, *r*) with confidence intervals, effect sizes, degrees of freedom and *P* value noted<br>*Give P values as exact values whenever suitable.*
☒	☐	For Bayesian analysis, information on the choice of priors and Markov chain Monte Carlo settings
☒	☐	For hierarchical and complex designs, identification of the appropriate level for tests and full reporting of outcomes
☒	☐	Estimates of effect sizes (e.g. Cohen's *d*, Pearson's *r*), indicating how they were calculated

Our web collection on statistics for biologists contains articles on many of the points above.

Software and code

Policy information about availability of computer code

Data collection	ImagingWin: V2.41a, Walz GmbH, Germany, https://www.walz.com/products/chl_p700/imaging-pam_ms/downloads.html<br><br>Chl a and P700 measurements: Dual-PAM-100 Software DualPAM V3.10, Walz GmbH, Germany, https://www.walz.com/products/chl_p700/dual-pam-100/downloads.html<br><br>Gas exchange and simultaneous measurements of chlorophyll a fluorescence parameters: GFS-3000 system (Walz, Effeltrich, Germany) equipped with an Arabidopsis Chamber (3010-A, Walz, Effeltrich, Germany)<br><br>Immunodetection analysis: Fusion, Version 15-18, Vilber Lourmat, Eberhardzell, Germany<br><br>Northern analyses: Typhoon Scanner Control v5.0.1, GE Healthcare<br><br>Protoplasts examination: Axio Imager fluorescence microscope (Zeiss)
Data analysis	Signal detection in immunodetection assays: Bio-1D, version 15.03, Vilber Lourmat, Eberhardzell, Germany<br><br>Transit peptide prediction: TargetP (https://services.healthtech.dtu.dk/service.php?TargetP-2.0)<br><br>Protein sequence alignments and the phylogenetic tree: CLC workbench software (version 20)

Protein structures: ChimeraX (https://www.rbvi.ucsf.edu/chimerax/)

Boxplots: BoxPlotR (http://shiny.chemgrid.org/boxplotr/)

Statistical analysis: R v3.5.2 (https://www.r-project.org/). Non-parametric Kruskal-Wallis tests were used, which were followed by pairwise Dunn's tests employing the R package dunn.test. P-values were adjusted on an experiment level using the Benjamini–Hochberg method.

Co-expression analysis: Atted-II (https://atted.jp/)

Functional profiling of AT3G48420 co-expressed genes (https://biit.cs.ut.ee/gprofiler/gost)

For manuscripts utilizing custom algorithms or software that are central to the research but not yet described in published literature, software must be made available to editors and reviewers. We strongly encourage code deposition in a community repository (e.g. GitHub). See the Nature Portfolio guidelines for submitting code & software for further information.

Data

Policy information about availability of data

All manuscripts must include a data availability statement. This statement should provide the following information, where applicable:

- Accession codes, unique identifiers, or web links for publicly available datasets
- A description of any restrictions on data availability
- For clinical datasets or third party data, please ensure that the statement adheres to our policy

The authors declare that all data presented in this study are available in the figures and the accompanying Supplementary Information file. The source data underlying Fig. 2 b-f; Fig. 3; Fig. 4; Fig 5; Fig. 6a, c; Supplementary Fig. 2b-c; Supplementary Fig. 3b; Supplementary Fig. 4; Supplementary Fig. 5; and Supplementary Table 2, as well as detailed corresponding statistics, are provided as a Source Data file. Other data that support the study are available from the corresponding author.

Human research participants

Policy information about studies involving human research participants and Sex and Gender in Research.

Reporting on sex and gender	*Use the terms sex (biological attribute) and gender (shaped by social and cultural circumstances) carefully in order to avoid confusing both terms. Indicate if findings apply to only one sex or gender; describe whether sex and gender were considered in study design whether sex and/or gender was determined based on self-reporting or assigned and methods used. Provide in the source data disaggregated sex and gender data where this information has been collected, and consent has been obtained for sharing of individual-level data; provide overall numbers in this Reporting Summary. Please state if this information has not been collected. Report sex- and gender-based analyses where performed, justify reasons for lack of sex- and gender-based analysis.*
Population characteristics	*Describe the covariate-relevant population characteristics of the human research participants (e.g. age, genotypic information, past and current diagnosis and treatment categories). If you filled out the behavioural & social sciences study design questions and have nothing to add here, write "See above."*
Recruitment	*Describe how participants were recruited. Outline any potential self-selection bias or other biases that may be present and how these are likely to impact results.*
Ethics oversight	*Identify the organization(s) that approved the study protocol.*

Note that full information on the approval of the study protocol must also be provided in the manuscript.

Field-specific reporting

Please select the one below that is the best fit for your research. If you are not sure, read the appropriate sections before making your selection.

[x] Life sciences [ ] Behavioural & social sciences [ ] Ecological, evolutionary & environmental sciences

For a reference copy of the document with all sections, see nature.com/documents/nr-reporting-summary-flat.pdf

Life sciences study design

All studies must disclose on these points even when the disclosure is negative.

Sample size	The sample size was based on previous experience and typical sample sizes described in the literature.
Data exclusions	n.a.
Replication	Fresh weight determination: 9-10 plants per genotype

Dual-PAM measurements: 5 plants per genotype

Gas exchange measurements: 3 plants per genotype

Imaging-PAM analysis: 5 leaves of 5 plants (25 data points)

Pigment analysis: leaf material of 5 individual plants per genotype

Phosphate release: MBP-AtCbbYA (4-7 technical replicates), MBP-AtCbbYB (4 technical replicates), CIP (2-3 technical replicates)

Enzyme kinetics: 3 technical replicates

Northern analyses were carried out once.

Immunodetection of AtCbbYA and AtCbbYB in cbby mutants: 3 replicates with independent biological replicates (a biological replicate indicates pooled leaves from 3-5 indiviual plants).

Immunodetection of RsCbbY-eGFP: 3 replicates with independent biological replicates (a biological replicate indicates pooled leaves from 3-5 indiviual plants).

Immunodetection of AtCbbYA-eGFP and AtCbbYB-eGFP in overexpressor lines: 2 replicates with independent biological replicates (a biological replicate indicates pooled leaves from 3-5 indiviual plants).

Fractionation and immunodetection of AtCbbYA/AtCbbYB were carried out once .

Immunodetction of Rubisco activase (Rca) and Rubisco: experiments were carried out three times with similar results. For each experiment proteins were extracted from pooled leaves of three individual plants.

Randomization	Arabidopsis plants were randomly placed in the growth chambers to prevent differences in light exposure, irrigation, and humidity.
Blinding	Blinding was not relevant, because the Arabidopsis double mutant atcbbyab showed already a distinct visible growth phenotype.

Reporting for specific materials, systems and methods

We require information from authors about some types of materials, experimental systems and methods used in many studies. Here, indicate whether each material, system or method listed is relevant to your study. If you are not sure if a list item applies to your research, read the appropriate section before selecting a response.

Materials & experimental systems

n/a	Involved in the study	
☐	☒	Antibodies
☒	☐	Eukaryotic cell lines
☒	☐	Palaeontology and archaeology
☒	☐	Animals and other organisms
☒	☐	Clinical data
☒	☐	Dual use research of concern

Methods

n/a	Involved in the study	
☒	☐	ChIP-seq
☒	☐	Flow cytometry
☒	☐	MRI-based neuroimaging

Antibodies

Antibodies used

The antibody against 6xHis-AtCbbYA66-319aa was generated in Roberto Barbato's laboratory; dilution of 1:1000

The antibody against AtCbbYB(47-316aa)-6xHis was generated by Pineda (Berlin, Germany) and subsequently purified by affinity chromatography with immobilized MBP-AtCbbYB; dilution of 1:1000

GFP-specific antibody (A-6455, ThermoFisher Scientific), dilution of 1:7500

Anti-Rca (Rubisco activase): Agrisera, AS10 700, dilution of 1:5000

Anti-RbcL: Agrisera, AS03 037, dilution of 1:5000

Anti-CoxII (cytochrome oxidase subunit II): Agrisera, AS04 053A, 1:1000

Anti-Lhcb3 (LHCII type III chlorophyll a/b-binding protein), Agrisera, AS01 002, 1:2000

Anti-Csp41b, provided by David Stern; dilution of 1:5000

Goat Anti-Rabbit IgG Antibody HRP-conjugate (Sigma-Aldrich A9169), batch number 015M4858V, used dilution 1:30,000

Validation

Anti-AtCbbYA and Anti-AtCbbYB were generated in this study as described in the Methods section. Antibodies were tested by immunodetection assays using leaf protein extracts isolated from plants lacking AtCbbYA, AtCbbYB or both proteins (see manuscript). Moreover, fusions of AtCbbYA-eGFP and AtCbbYB-eGFP were immunodetected in stably transformed overexpressor plants (see manuscript).

Antibodies obtained from Agrisera (https://www.agrisera.com/). Validation is provided on the following web pages:
Anti-Rca, https://www.agrisera.com/en/artiklar/rubisco-activase.html
Anti-RbcL, https://www.agrisera.com/en/artiklar/-rbcl-rubisco-large-subunit-form-i-rabbit.html
Anti-CoxII, https://www.agrisera.com/en/artiklar/coxii-cytochrome-oxidase-subunit-ii-marker-of-mitochondrial-inner-membrane.html
Anti-Lhcb3, https://www.agrisera.com/en/artiklar/lhcb3-lhcii-type-iii-chlorophyll-a_b-binding-protein.html

Anti-CSP41b was provided by David Stern [Bollenbach, T. J., Sharwood, R. E., Gutierrez, R., Lerbs-Mache, S. & Stern, D. B. The RNA-binding proteins CSP41a and CSP41b may regulate transcription and translation of chloroplast-encoded RNAs in Arabidopsis. Plant Mol. Biol. 69, 541–552 (2009)] and was also validated and employed in Qi et al. [Qi, Y. et al. Arabidopsis CSP41 proteins form multimeric complexes that bind and stabilize distinct plastid transcripts. J. Exp. Bot. 63, 1251–1270 (2012)].
